# Polygenic and monogenic adaptation drive evolutionary rescue at different magnitudes of environmental change

**DOI:** 10.1093/jhered/esag026

**Published:** 2026-04-07

**Authors:** Tatiana Bellagio, Moises Exposito-Alonso

**Affiliations:** Department of Integrative Biology, University of California Berkeley, Berkeley, CA, United States; Howard Hughes Medical Institute, University of California Berkeley, Berkeley, CA, United States; Department of Plant Biology, Carnegie Institution for Science, Stanford, CA, United States; Department of Biology, Stanford University, Stanford, CA, United States; Department of Integrative Biology, University of California Berkeley, Berkeley, CA, United States; Howard Hughes Medical Institute, University of California Berkeley, Berkeley, CA, United States; Department of Plant Biology, Carnegie Institution for Science, Stanford, CA, United States; Department of Biology, Stanford University, Stanford, CA, United States

**Keywords:** evolutionary rescue, polygenic adaptation, genetic architecture, predictability of adaptation, environmental change

## Abstract

Understanding the genetic basis of rapid adaptation is key to predicting species’ evolutionary responses to environmental change. However, it is still debatable whether many small-effect mutations or a few large-effect mutations underlie rapid adaptation, and how this knowledge can predict population survival or extinction. To address this question, we performed a series of ecologically grounded forward-in-time genetic simulations to study rapid adaptation and extinction with increasing magnitudes of environmental change. These simulations were seeded with genomic variation of the plant *Arabidopsis thaliana* to have a realistic genomic structure, with one (monogenic) to 1,000 (polygenic) variants with varying heritabilities contributing to an environmental adaptive trait. Our results revealed two distinct scenarios of rapid adaptation and population rescue. Under small-to-moderate environmental shifts, high polygenic traits increased evolutionary rescue probability. Under extreme environmental shifts, high polygenic traits lead predictably to extinction, yet monogenic traits sometimes produce one-off winning adaptive genotypes. We interpret our rapid evolutionary rescue findings in terms of the fundamental theorem of natural selection, where trait polygenicity shapes the distribution of genetic variance in fitness across replicates and, in turn, the probability of population survival, with polygenic architectures producing more stable and predictable fitness variance and monogenic architectures generating highly skewed and variable outcomes. These results highlight the insights genomics gives us into the (un)predictability of species’ evolutionary responses to global change, with management implications for assisted adaptation and conservation.

## Introduction

When environmental changes displace species from their ecological niches, they must either track suitable conditions through range shifts, adapt to novel environments, or face local to global extinction (Parmesan 2006; Wiens 2016; de Lafontaine et al. 2018). Populations’ adaptive capacity—driven by natural selection acting on within-species genetic variation—has become a critical survival determinant in a rapidly changing world (Thompson 1998; Barrett and Schluter 2008; IPCC 2022). Contrary to the classical view of evolution as a slow process, empirical studies in the genomics era have now demonstrated that rapid, genetically based phenotypic changes are widespread. Examples include industrial melanism in peppered moths (Cook and Saccheri 2013); insecticide resistance in *Drosophila* sp. evolving within a few decades of insecticide application (Daborn et al. 2002); climate-driven shifts in flowering time in *Brassica rapa*, observed over just a few generations (Franks et al. 2007); and widespread herbicide resistance in agricultural weeds (Heap 2024). Critically, such evolutionary dynamics may have consequences at the demographic level and, in cases of abrupt or extreme environmental change, may rescue a population from decline and extinction—achieving evolutionary rescue (Gomulkiewicz and Holt 1995; Bell and Gonzalez 2009; Carlson et al. 2014). Empirical evidence of evolutionary rescue is accumulating in both experimental and natural systems—microbial populations adapting to antibiotics or salinity stress (Bell and Gonzalez 2011; Lindsey et al. 2013), guppies colonizing novel environments (Carlson et al. 2014; Fitzpatrick et al. 2016) and plant common gardens across climates to evaluate the limits of evolutionary rescue (Wu et al. 2026). Such experiments are especially important because evolutionary responses might be insufficient to prevent population decline under increasingly severe or variable environmental changes (Hamann et al. 2018). Yet, the likelihood of rescue depends on multiple interacting factors, including population size, standing genetic variation, migration, and the rate or magnitude of environmental change (Carlson et al. 2014; Bell 2017; Souza et al. 2023). Despite this growing body of work, the genomic basis that enables some populations to persist but others go extinct remains poorly understood.

Whether populations can adapt depends not only on the presence of within-species genetic variation, but on its genetic architecture: the number, effect sizes, and interactions among adaptive loci across the genome, including complex phenomena such as dominance, pleiotropy, and epistasis. Among these dimensions, a long-standing question in evolutionary biology concerns whether populations typically evolve through the cumulative effects of many small-effect loci or a few large-effect loci (Orr 2005; Connallon and Hodgins 2021). Empirical evolutionary studies have identified prominent examples of well-described large-effect loci involved in adaptation, such as *CORTEX* driving industrial melanism in the peppered moth (Sheehan et al. 2016; Van’t Hof et al. 2016) and *YUP* influencing flower color transitions in *Mimulus* sp. (Liang et al. 2023), among many others (Dorweiler et al. 1993; Cooley et al. 2011; Smith and Rausher 2011). Concurrently, quantitative genomics literature has reported thousands of small-effect loci underlying many important traits across species (Khaipho-Burch et al. 2023). Examples of such traits are human height, where the number of identified loci continues to grow with increasing sample size (Boyle et al. 2017; Visscher et al. 2017), or grain yield traits in plants (Zaw et al. 2019; Cao et al. 2020). Regardless of the specific genetic architectures of adaptive traits across species, we still lack a clear understanding of when different architectures may benefit or prevent evolutionary rescue in natural populations.

The study of different genetic architectures and their influence on adaptation has typically involved theory and simulations with a focus on adaptation temporal dynamics. Much of the early work, rooted in classical population genetics, focused on understanding how beneficial alleles of large-effect sweep to fixation (Smith and Haigh 1974; Kaplan et al. 1989; Hermisson and Pennings 2005; Barrett and Schluter 2008). In contrast, work on polygenic adaptation was initially studied through the infinitesimal model, which treats traits as continuous, does not explicitly track individual loci, and assumes that additive genetic variance remains constant through time (Lande 1976; Walsh and Lynch 2018). More recent theoretical and simulation-based studies have modeled shifts in allele frequencies across many small-effect loci, increasingly incorporating biologically realistic factors such as demography, effect size distributions, and environmental change (Jain and Stephan 2017; Barton and Etheridge 2018; Barghi et al. 2020; John and Stephan 2020; Hayward and Sella 2022). To bridge the gap between monogenic and polygenic adaptation, recent work has explored conditions under which one architecture is favored over the other. These include redundancy and initial allele frequency (Höllinger et al. 2019), the magnitude of environmental change (Milligan et al. 2025), and selection spatial structure (Yeaman 2022) to explain when one or the other architecture will more likely emerge. However, most of these studies focus on scenarios where adaptation is assumed to occur, often explaining outcomes retrospectively in populations that, by definition, have adapted, which incurs a survival bias by not analyzing conditions when populations go extinct.

While genetic architecture has long been recognized as a key determinant of eco-evolutionary dynamics (Yamamichi 2022), its role in shaping the likelihood and predictability of evolutionary rescue remains poorly understood. Gomulkiewicz and Holt theoretically demonstrated that evolutionary rescue should be possible under both single-locus and quantitative models, assuming high initial genetic variance and moderate selection (Gomulkiewicz and Holt 1995). Building on this, (Orr and Unckless 2008) argued that polygenic architectures should offer greater potential for rescue due to their genetic redundancy, allowing adaptive allele frequency shifts at many loci without relying on rare, large-effect mutations. However, subsequent work by Gomulkiewicz et al. demonstrated through analytical models and simulations that increasing the number of loci underlying fitness can dilute the strength of selection acting on each locus, thereby slowing the rate of adaptation and narrowing the conditions under which populations can persist after an environmental shift (Gomulkiewicz et al. 2010). More recently, Kardos and Luikart showed through deterministic models and stochastic simulations that polygenic trait architectures—by maintaining higher short-term evolutionary potential—can substantially improve population viability during rapid environmental change compared with traits controlled by few large-effect loci (Kardos and Luikart 2021).

To clarify this unresolved question, we conducted a large-scale set of forward-in-time population genetic simulations to test how genetic architecture influences the probability of evolutionary rescue under very rapid environmental change within 10 generations. We modeled a quantitative trait under varying levels of polygenicity and heritability, and tracked population outcomes across different magnitudes of environmental change. By explicitly comparing architectures ranging from few large-effect to many small-effect loci, we examined how genetic architecture shapes both the likelihood and predictability of evolutionary rescue. To ensure biological relevance, our simulations were grounded in empirical genomic data from *Arabidopsis thaliana*, a model system in eco-evolutionary dynamics (Leventhal et al. 2025a).

## Materials and methods

### Simulations

We focus on modeling rapid adaptation from standing genetic variation, as the pace of global change may require plant and animal species to rely on pre-existing diversity (Barrett and Schluter 2008). To simulate realistic genomic architectures, we used genome-wide variation data from a diverse founder population, which included 10 individuals from 231 different *A. thaliana* accessions variable in 3,235,480 Single Nucleotide Polymorphisms (SNPs), mimicking empirical experimental evolution in outdoor gardens of this species (GrENE-net.org project) (Wu et al. 2026). As a model system in eco-evolutionary dynamics, *A. thaliana* provides measured allele-frequency spectra, linkage disequilibrium (LD) decay, and a diverse genomic panel, allowing us to build simulations based on an empirical genome structure. At the same time, its predominantly selfing mating system—with limited but nonzero outcrossing, as in many plant species—increases LD and interference among beneficial variants. Accordingly, our quantitative evolutionary rescue rates should be viewed as conservative, likely slower and more stochastic than those for highly outcrossing species. To load genome-wide variation in population genetic simulations with SLiM (Kelleher et al. 2018; Haller et al. 2019), we implemented a tree sequences approach (Kelleher et al. 2019), where neutral mutations were initially removed and represented by the ancestral recombination graph so only trait-relevant mutations were monitored through the simulations.

**Fig. 1:**
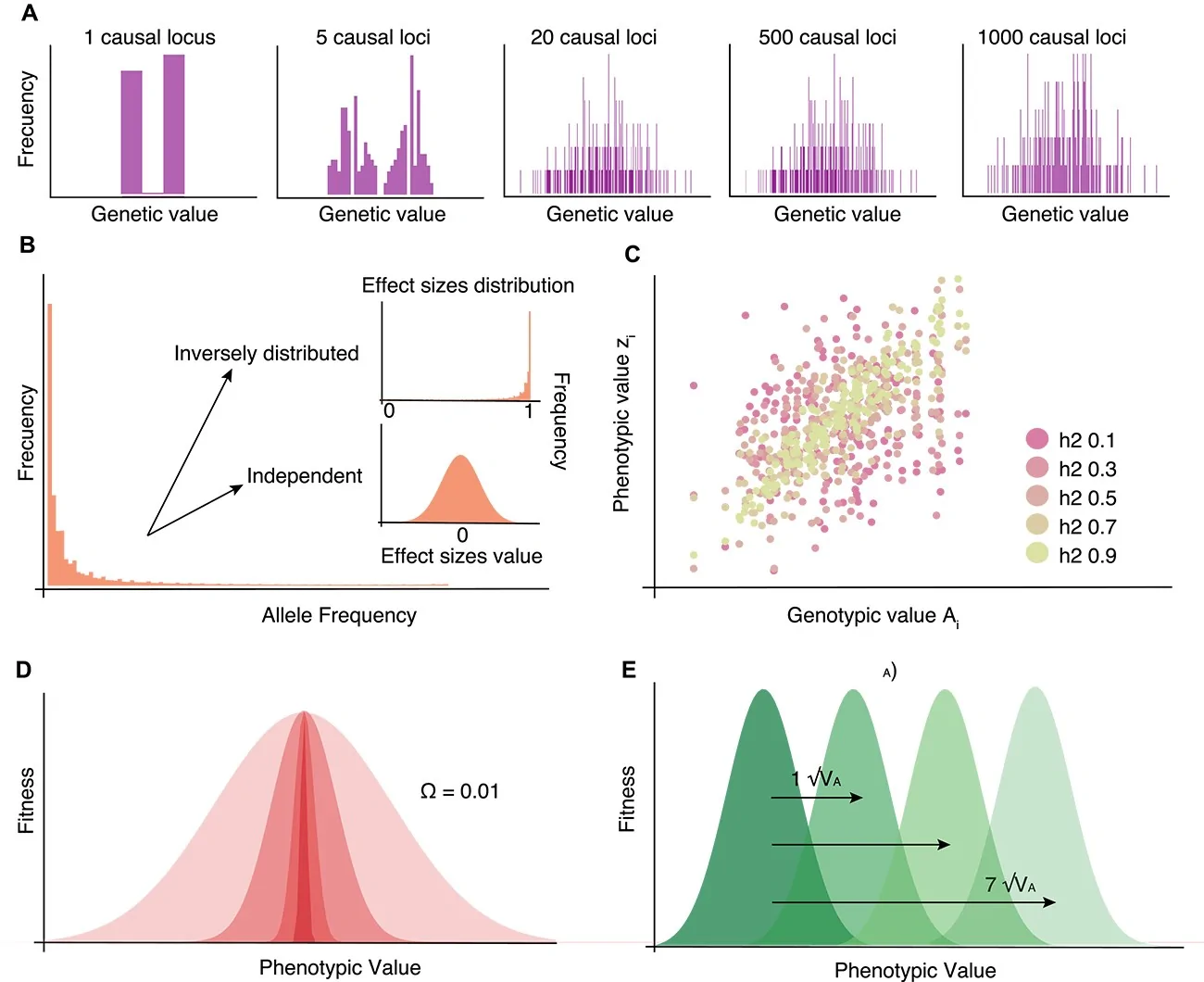
Parameter space. (A) Resulting genetic values distributions, with 5 of the 9 polygenicity levels shown for visualization. (B) Initial allele frequency taken from the founder population site frequency spectrum, effect sizes of the contributing loci taken from a distribution inverse to the initial allele frequency (dependent) or from a normal distribution with mean 0 and standard deviation 2 (independent), (C) Heritability levels, (D) Selection width modeled as fitness decay with *Ω* = 0.01, and (E) Distance to the new phenotypic optimum modeled as standard deviations from initial genetic values mean *Ā*_0_ with 3 of the 8 shifts shown for visualization.

We modeled a single adaptive trait under natural selection. The trait’s genetic architecture varied in three parameters: polygenicity, initial allele frequency, and effect size distribution. Polygenicity ranged across nine levels (1 to 1,000 loci; Fig. 1). Initial allele frequencies were sampled from the *A. thaliana* site frequency spectrum. Effect sizes were either drawn from a normal distribution *N* ≈ (0, 2) or inversely proportional to allele frequency. This latter approach reflects a well-supported trade-off between allele frequency and effect size, where theoretical and empirical studies indicate that phenotypes historically shaped by stabilizing selection tend to maintain large-effect alleles at lower frequencies, but alleles at higher frequencies typically have smaller effects, reflecting an inverse relationship between allele frequency and effect size (Simons et al. 2018, 2025). It is worth noticing that this assumption does not necessarily extend to traits shaped by other forms of selection, like balancing selection where large-effect loci are commonly observed (Prince et al. 2017; Groh et al. 2025). Since both approaches produced the same qualitative patterns of evolutionary rescue, we focus on the empirically-based inverse model in the main results. We assume that allelic effects on the trait combine in an additive manner: *A_i_* = *∑X_ij_a_j_*, where *A_i_* is the trait’s genetic value of the individual *i*, *X_ij_* is the individual’s genotype at locus *j*, and *a_j_* is its effect size. The phenotypic additive genetic variance, *V_A_* is then defined as the variance in *A_i_* across the population. *A_i_* were standardized in the first generation to ensure comparability across simulations, such that *μ* = 0, *σ*^2^ = 1. We simulated the trait across five target narrow-sense heritability levels (0.1 to 0.9) under an additive, single-environment model. Under these assumptions: *h*^2^ = *V_A_/V_A_* + *V_E_*. For each target *h*^2^, we initialized the environmental variance as *V_E_* = (*V_A_* − *h*^2^*·V_A_*)/*h*^2^. This *V_E_* value was then held constant throughout the simulation, such that environmental noise *en_i_* for each individual was sampled from the same *N*(0, *V*_E_) distribution throughout time, and phenotypes were calculated as *z_i_* = *A_i_* + *en_i_* (Fig. 1). See Table 1 for notations.

**Table 1:** Notation table.

*A_i_*	Additive genetic value of individual *i* for the trait = *∑X_ij_a_j_*
*V_A_*	Population additive genetic variance of the trait = *V_A_*(*z*)
*h* ^2^	Narrow sense heritability = *V_A_/V_A_* + *V_E_*
*en_i_*	Environmental noise for individual *i* sampled from *N*(0, *V_E_*)
*z_i_*	Phenotypic value of individual *i* = *A_i_* + *en_i_*
*w_i_*	Fitness of individual *i* = exp(−(0.5 × (*z_i_ − Z*_opt_)^2^)/*Ω*)
*V_A_*(*w*)	Population additive genetic variance in fitness
*Z*_opt_	Population new environmental optimum defined in units of starting standardized genetic values deviation *$\surd{} $V_A_*
*∆*_shift_	Magnitude of environmental shift = *Ā* _0_ *− Z*_opt_
*Ω*	Variance parameter controlling the width of the Gaussian fitness function

To model environmental change, we simulated eight levels of phenotypic optimum shifts (i.e. environmental change), measured in standard deviations from the initial population *A_i_* mean (*Ā*_0_) (Fig. 1). Viability selection was applied via the stabilizing selection model, where individual fitness (*w_i_*) represents survival probability and was calculated as *w_i_* = exp(−(0.5 × (*z_i_* − *Z*_opt_)^2^)/*Ω*) where *Z*_opt_ is the new phenotypic optimum and *Ω* represents the variance parameter controlling the width of the Gaussian fitness function. We conducted simulations at different magnitudes of environmental shift: *∆*_shift_ = *Ā*_0_ − *Z*_opt_; which we measure in units of starting standardized genetic values deviation $\surd{} $*V_A_*. We tested a range of scenarios varying in Gaussian selection strength (*Ω*) and environmental shift (*∆*_shift_), and ultimately set these parameters to *Ω* = 0.01 and *∆*_shift_ = 1 to 7, which would produce outcomes spanning from complete survival to total extinction. This parameter combination was also necessary to recreate classic evolutionary rescue dynamics, in which a sudden and substantial environmental shift causes an initial decline in population size, followed by either recovery or collapse (Fig. 5c) (note that this corresponds to dynamics of environmental shifts beyond what; Milligan et al. 2025).

To infuse simulations with biological realism analogous to *A. thaliana* experiments, we conducted simulations after demographic observations from field garden observations (Wu et al. 2026). This included a starting population size of 2,310 individuals and density-dependent population regulation via a fixed carrying capacity, such that at the end of each generation individuals were randomly sampled to maintain the population at 900 which matches the maximum population sizes observed in outdoor experiments (Wu et al. 2026). Furthermore, we used an empirical recombination rate of ≈4 cM/Mb (Singer et al. 2006). Generations were annual, nonoverlapping, and assumed a 97% selfing rate (Platt et al. 2010). Offspring production followed a Poisson distribution with a mean of 7.247, representing the number of successfully recruited offspring per parent after seed dispersal and early mortality. This value was derived from our field observations (Leventhal et al. 2025a) and is on par with other common gardens (Ågren and Schemske 2012; Picó 2012). Selection acted solely through individual survival probabilities determined by the Gaussian fitness function, so only surviving individuals contributed offspring to the next generation.

For each level of polygenicity (×9), we generated 30 independent genetic architectures (×30), which were combined with five heritability levels (×5) and seven environmental shift scenarios (×7). Each unique combination was replicated five times (×5) to account for stochasticity in the simulations. In total, we performed 47,250 forward-in-time genetic simulations, each of them tracking a single population for 10 generations. At each generation, we extracted key data including population size, magnitude of environmental shift, individual genotypes, phenotypes, and fitness values, in addition to saving full tree sequences for downstream analysis. After 10 generations, each simulated population was classified as either “survived” (persisted to generation 10) or “went extinct” (population size = 0). These outcomes were used to model population survival probability in subsequent analysis. All statistical analyses were conducted in Python. Linear, multiple, and logistic regression models were implemented using the Statsmodels library, and decision tree classifiers were implemented using Scikit-learn.

Simulations were implemented in a reproducible Snakemake pipeline (Mölder et al. 2021) https://github.com/Tatianabellagio/slim_grenenet/tree/es_dep_af, which takes the starting VCF, runs all simulation combinations, and produces summary outputs.

### Empirical data

To explore intuitions of phenotypic shifts in polygenic and monogenic cases, we visualized a pair of recent *A. thaliana* datasets.

For each experimental field site in Wu et al. (2026), we compiled the population fate across replicate plots (survived vs. extinct), the site’s climate (mean annual temperature), and the ecotype composition with starting and “evolved populations” frequencies. From the published frequencies, we estimated a site-level heritability of fitness as the fraction of variance in frequency change attributable to ecotype identity (among-ecotype variance/total variance). Assuming that ecotypes are distributed along an environmental gradient and that their fitness is maximized around the climatic mean of their native distributions, we expressed each experimental garden’s climate as a deviation from that putative phenotypic optimum ($\surd{} $*V_P_*) (Fig. 8).

We re-plotted flowering time for wild-type (wt) and respective *flc* knockout *A. thaliana* lines (47 + 47) from Ruffley et al. (2024) under long-day (22°C) conditions. For each genotype, we averaged flowering time across three replicates per ecotype. The change in mean flowering time of the *flc* knockout was expressed in units of standard deviations of total phenotypic variance ($\surd{} $*V_P_*) relative to the initial wild type mean (Fig. 9).

## Results

### Simulation parameters defining population mortality versus evolutionary rescue

**Fig. 2:**
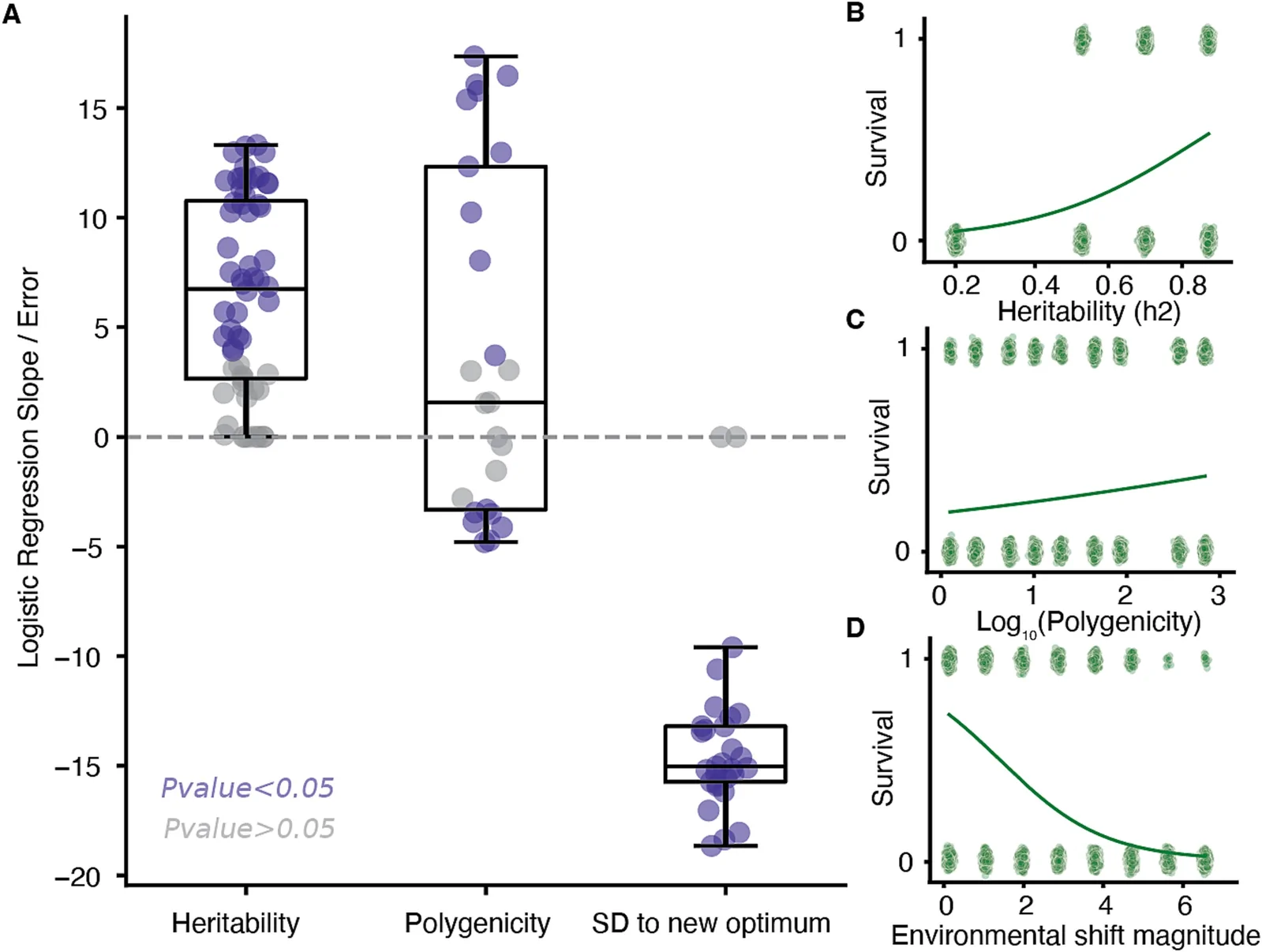
Effects of genetic architecture and environmental change on population survival. (A) Distribution of standardized logistic regression slopes (*z*-values) for models predicting survival across simulation parameters. Each boxplot shows the distribution of standardized effect sizes from logistic regressions of survival on each parameter, while holding the other parameters constant at simulation onset. (b–d) Effects of (B) heritability, (C) polygenicity, and (D) the magnitude of environmental shift on population survival across 43,200 simulations. Survival probability declined sharply with increasing environmental change (*z* = −92, *P* < 1 × 10^−2^), while heritability had a strong positive effect (*t* = 80, *P* < 1 × 10^−2^). Polygenicity also had a small but statistically significant positive effect (*z* = 23, *P* < 1 × 10^−2^).

To examine how heritability, polygenicity, and the magnitude of environmental change affect the ability of a population to adapt and persist, we first performed a multiple logistic regression across all simulations. As expected, populations’ survival probability declined sharply with increasing environmental change (*z* = −92, *P* < 1 × 10^−2^, *n* = 43,200, Fig. 2d), but heritability had a strong positive effect on populations’ survival (*t* = 80, *P* < 1 × 10^−2^, *n* = 43,200, Fig. 2b). Notably, polygenicity also had a small but statistically significant positive effect (*z* = 23, *P* < 1 × 10^−2^, *n* = 43,200, Fig. 2c).

Given the modest effect size of polygenicity in population survival, we hypothesized that the influence of polygenicity on survival might be context-dependent. To explore this, we ran independent logistic regressions within fixed combinations of polygenicity, heritability, and environmental shift, allowing us to isolate the effect of a single parameter on survival but holding all others constant—such that variation across replicates could be attributed to that parameter and to stochasticity from selection and drift (Fig. 2). This approach confirmed the consistent positive role of heritability (*z* = 3.9 to 13.3, *P* < 9 × 10^−5^, *n* = 40), and the strongly negative impact of environmental change (*z* = −18.7 to −9.6, *P* < 8 × 10^−22^, *n* = 27). However, the relationship of polygenicity with population rescue spanned both positive and negative ranges (*z* = −4.8 to 17.4, *P* < 9 × 10^−4^, *n* = 17), confirming that the effect of polygenicity on survival depends on the specific selection and genetic context.

### Polygenicity has context-dependent effects on evolutionary rescue

**Fig. 3:**
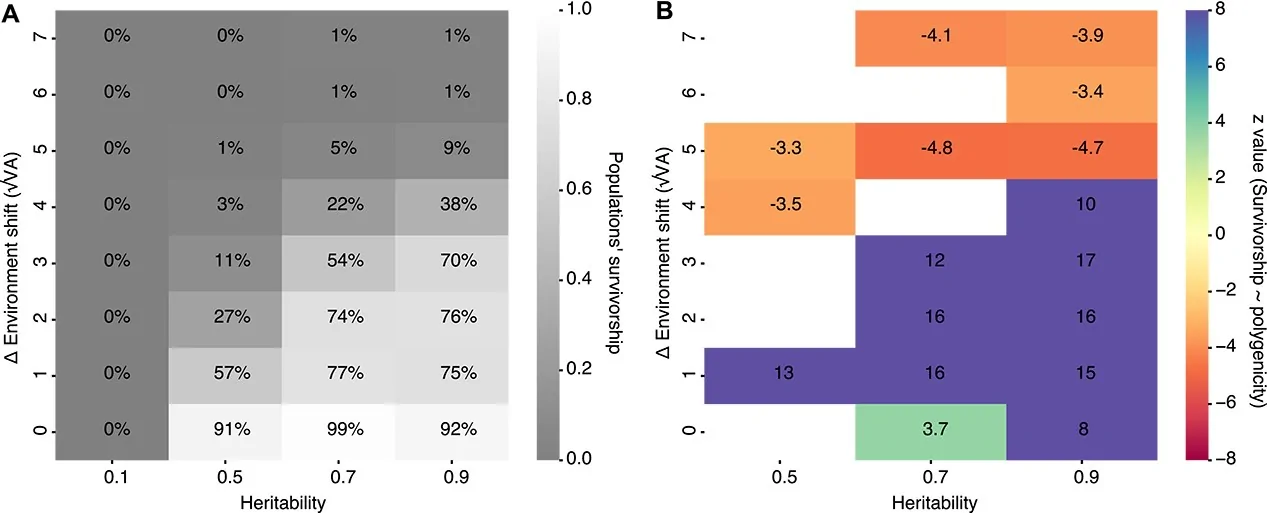
Impact of polygenicity on population survival after the 10th generation. (A) Survival rates across combinations of heritability and environmental shift. (B) *z* Values from logistic regressions showing the effect of polygenicity on survival, for each parameter combination.

We further explored the combination of parameters that contributed to either a positive or negative effect of polygenicity on population survival, and observed that increased polygenicity had a positive impact on population survival under low-magnitude environmental changes (*∆*_shift_  $\surd{} $*V_A_* to new optimum = 0 to 3; *z* = 3.7 to 17.4, *P* < 2 × 10^−4^) where the overall population survivorship was high (range = 38% to 99%). Conversely, the opposite effect was observed under large environmental changes (*∆*_shift_ = 5 to 7; *z* = −4.8 to −3.3, *P* < 9 × 10^−4^) where low-polygenic architectures led to increased survival, although overall population survival was extremely low (1% to 9%) (Fig. 3).

### Heterogeneity in genetic variance in fitness as a predictor of population survival

**Fig. 4:**
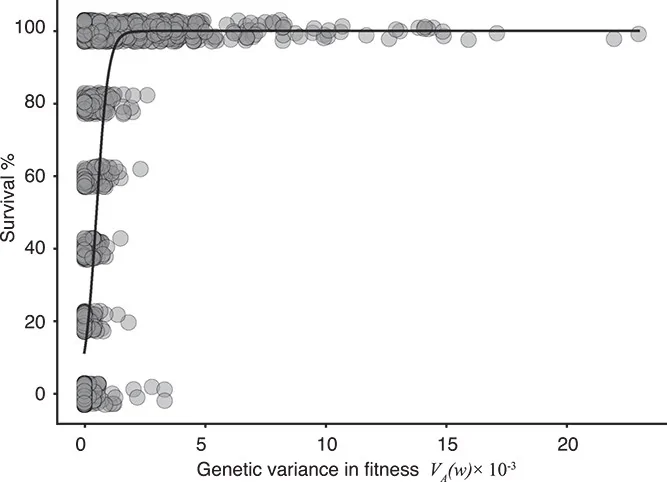
Relationship between initial additive genetic variance in fitness *V_A_*(*w*) and population survival on the last generation. Each point represents a unique parameter combination with survival expressed as the percentage of replicates (out of 5) in which the population persisted. All results shown for simulations initialized with heritability = 0.7.

**Fig. 5:**
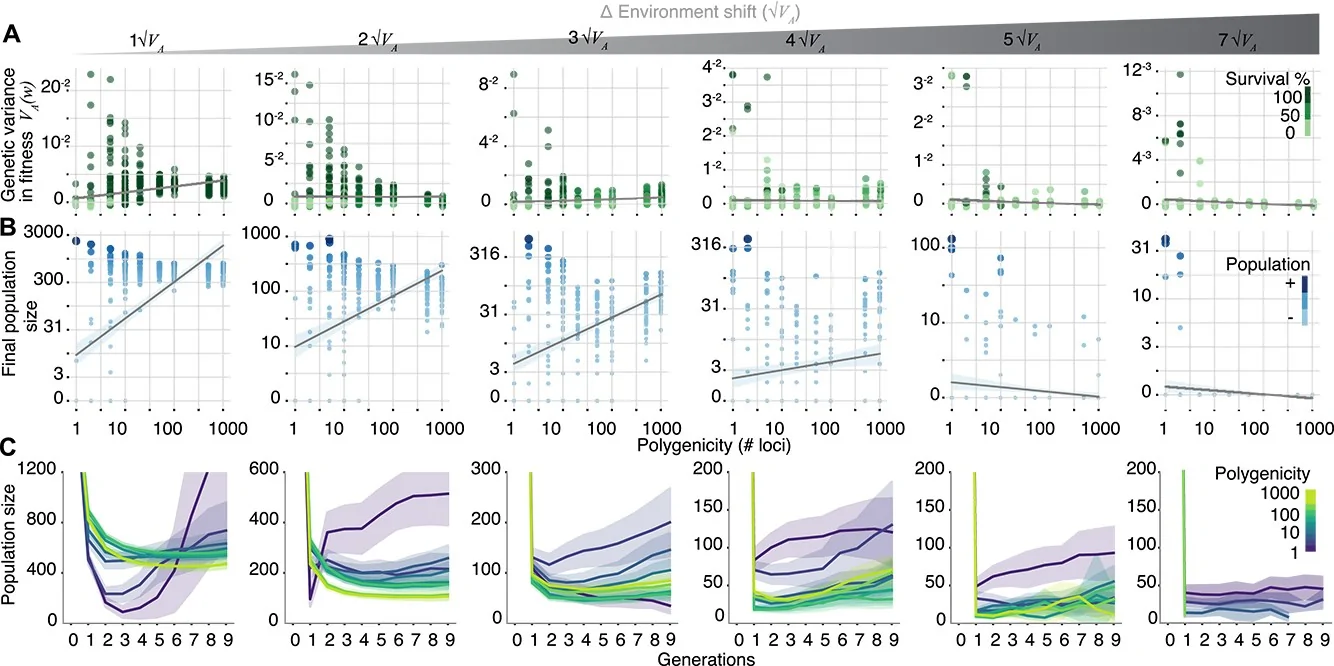
Effects of polygenicity on population genetics and demography under varying magnitudes of environmental change. (A) The effect of polygenicity on additive genetic variance in fitness (*V_A_*(*w*)) changes under different magnitudes of environmental change. Under small environmental shifts (1 to 2 *∆*_shift_), *V_A_*(*w*) increased significantly with greater polygenicity (*P* < 0.05). This relationship weakened and became nonsignificant under larger shifts (3 to 7 *∆*_shift_). (B) Relationship between polygenicity and final population size (10th generation) under different magnitudes of environmental change. Low-polygenic architectures exhibited greater variance in final population size across all magnitudes of environmental change, with some replicates achieving the largest population sizes. High-polygenic architectures showed more consistent demographic outcomes that closely mirrored survival probabilities. Positive associations occurred at small shifts (1, 2, and 3 *∆*_shift_  *P* < 0.05), but these weakened and became nonsignificant under larger shifts (4 to 7 *∆*_shift_). (C) Population trajectories across time under different polygenic architectures and environmental shift magnitudes. All simulations began with an initial population size of 2,310. When survival occurred, populations typically exhibited U-shaped demographic trajectories characteristic of evolutionary rescue. All results shown for simulations initialized with heritability = 0.7, columns show results for *∆*_shift_ = 1, 2, 3, 4, 5, and 7, while results for the remaining shift magnitude are presented in Supplementary Figs S3–S5.

We observed that genetic variance in fitness, *V_A_*(*w*), varied across replicates even when simulations shared the same genetic architecture, and varied substantially across genetic architectures despite all having the same starting phenotypic genetic variance *V_A_*. Randomness in the sampled allelic effects and number of contributing loci introduced differences in *V_A_*(*w*). Supporting the long-standing theory that *V_A_*(*w*) reflects adaptive potential (Fisher 1930), we found as expected a strong positive relationship between initial *V_A_*(*w*) and population survival across all scenarios (*z* = 18.47, *P* < 1 × 10^−2^, considering simulations initialized with *h*^2^ = 0.7, Fig. 4). To better understand the context-dependent effects of polygenicity on survival identified earlier, we then explored how polygenicity influences the mean and variability of *V_A_*(*w*) under a gradient of *∆_shift_* environmental change (Fig. 5a, simulations initialized with *h*^2^ = 0.7). Across this gradient, we observed a consistent pattern: under mild environmental change *V_A_*(*w*) increased significantly with polygenicity (*∆_shift_* < 2, *P* < 1 × 10^−2^), supporting higher survival probabilities in highly polygenic architectures. Under large environmental changes, the relationship inverted: higher average *V_A_*(*w*) was associated with lower polygenicity (*∆*_shift_ > 3, *P* < 1 × 10^−2^). This pattern helps explain why low-polygenic architectures sometimes outperformed, on average, highly polygenic ones under extreme environmental shifts, despite greater variance in survival outcomes.

To further explore how polygenicity influences demographic outcomes, we examined both the final population sizes (Fig. 5b) and temporal trajectories (Fig. 5c) across simulation replicates. Despite the overall trend that low-polygenic architectures were associated with lower survival probabilities, we observed that, conditional on survival, monogenic architectures led to larger final population sizes compared with high-polygenic populations across all levels of environmental change. Furthermore, low-polygenic populations exhibited greater variability in final size, likely driven by highly variable fitness variance (*V_A_*(*w*)). In contrast, high-polygenic architectures showed much less variability: population sizes tightly mirrored survival trends, with stable populations under small environmental shifts and sharp declines toward extinction under severe shifts.

**Fig. 6:**
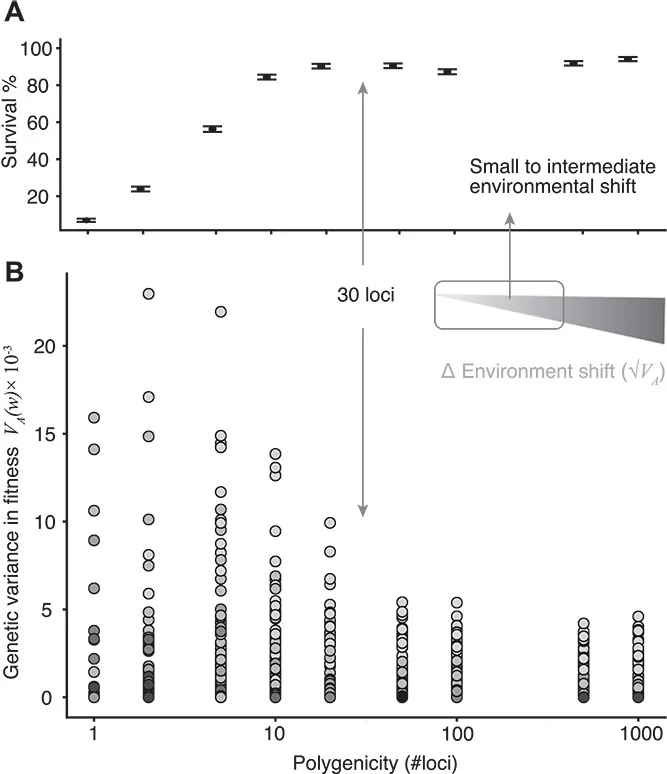
Relationship between polygenicity, population survivorship, and fitness variance under different magnitudes of environmental change. (A) Population survivorship across levels of polygenicity under small-to-intermediate environmental shifts. More polygenic architectures consistently show higher probabilities of population survival. (B) Lower-polygenic architectures exhibit significantly greater variability in *V_A_*(*w*) compared with highly polygenic ones. A sharp decline in *V_A_*(*w*) variability and increased population survival under small-to-intermediate environmental change occurs around 30 contributing loci.

To explain the counterintuitive relationship of *V_A_*(*w*) and polygenicity, we studied the variation in *V_A_*(*w*) across simulations. We found that the variability of *V_A_*(*w*) across replicates was consistently greater in low-polygenic architectures, regardless of the magnitude of environmental change. Using multiple regression while controlling for heritability at simulation onset and environmental shift, we confirmed that lower polygenicity was associated with significantly higher variability *sd*(*V_A_*(*w*)) across simulation replicates (polygenicity *t* = −5.496, *P* < 1 × 10^−2^) and higher skew under moderate environmental change (*Skew*[*V_A_*(*w*)]_oligo_/*Skew*[*V_A_*(*w*)]_poly_ = 2.1). Furthermore, the variability in *V_A_*(*w*) across simulations decayed rapidly around 30 contributing loci (Fig. 6), suggesting a potential threshold separating architectures with stochastic versus more predictable evolutionary dynamics, coinciding with the central limit.

### Predictability of evolutionary rescue under monogenic and polygenic architectures

**Fig. 7:**
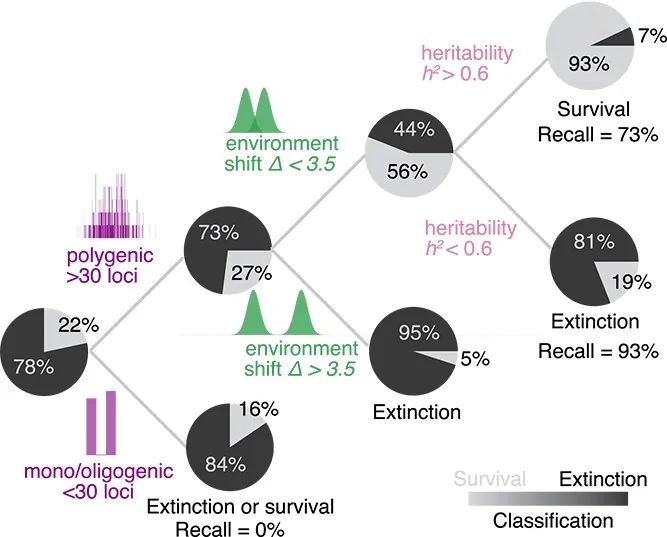
Decision tree models trained to predict population survival based on heritability, polygenicity, and the magnitude of environmental change. Two separate models were trained: One for low-polygenic architectures (<30 loci) and one for high-polygenic architectures (>30 loci). At each node, pie charts show the proportion of simulations classified as survived or extinct. Branch splits reflect thresholds learned by the model during training.

Building on the observation that fitness variance, *V_A_*(*w*), is more volatile under low-polygenic architectures than polygenic architectures (Fig. 6), we asked whether this variability could impact the predictability of evolutionary rescue. To investigate this, we categorized simulations into low- and high-polygenic groups based on the number of contributing loci, using 30 loci as a threshold identified from the decay in *V_A_*(*w*) variability. Within each group, we trained a decision tree classifier (depth = 2) using a 70 to 30 train-test split, with heritability, polygenicity, and environmental shift magnitude as predictors (Fig. 7). For high-polygenic architectures (>30 loci), survival was highly predictable: the decision tree correctly classified 91% of simulations in the test set. In this model, the primary predictor of survival was the magnitude of environmental shift (feature importance = 0.52), with a threshold at 3.5 $\surd{} $*V_A_* separating surviving and nonsurviving populations. In contrast, for low-polygenic architectures (<30 loci), survival was unpredictable. In fact, the model always predicted extinction under monogenic architectures, and this was often successful because monogenic architecture simulations led to 84% extinction; the model, however, recalled zero survival cases. This indicates that the model did not find useful heritability or environmental shift information as predictors. On the other hand, in high-polygenic architectures, heritability was the second most important predictor (threshold *h*^2^ = 0.3), but polygenicity itself (very large vs. moderately large number of loci) had no predictive value. Overall, these findings suggest that low-polygenic architectures generate nonpredictable survival outcomes. They further support the existence of a tipping point near 30 contributing loci, beyond which evolutionary outcomes become more deterministic and less sensitive to the underlying genetic architecture, including stochastic differences in initial allele frequencies and effect sizes.

## Discussion

Our study reveals that the effectiveness of different genetic architectures in promoting evolutionary rescue depends critically on the magnitude of environmental change. We found that polygenic architectures—those involving many small-effect loci—consistently enhanced survival under mild-to-moderate environmental shifts, but low-polygenic architectures were more likely to lead to extinction but exceptionally enabled rescue under severe environmental change. This transition arises because polygenic traits provide smoother, predictable adaptive responses when the required phenotypic shift is small, whereas large-effect alleles can produce the rare, extreme phenotypes necessary for survival when environments shift beyond the existing phenotypic range. In nature, there are abundant examples of adaptation occurring either through polygenic or monogenic architectures (Bomblies and Peichel 2022), a distinction that may often be idiosyncratic to the ecology of species and its environment. Perhaps then the key question in the longstanding “many vs few genes” debate is not whether polygenic or monogenic architectures may be more or less common, but rather under which environmental conditions each genetic architecture is most likely to fuel rapid evolutionary rescue.

Recent studies have begun to clarify the ecological and genetic factors that influence whether adaptation is driven by a few genes of large effect or many genes of small effect. Höllinger et al. emphasized that monogenic and polygenic architectures are all but the same with the key distinction being simply the amount of background loci in the genome that redundantly provide similar paths to adaptation (Höllinger et al. 2019). Yeaman et al. further showed that the type of selection acting on a trait—directional vs spatially-varying—can influence the emergence of different architectures, with spatially-varying selection tending to favor fewer, larger-effect loci, and directional selection favoring more polygenic responses (Yeaman 2022). Most recently, Milligan et al. showed that polygenic adaptation tends to dominate under moderate environmental shifts, but large-effect alleles are more likely to fix faster and contribute more to fitness gains when environmental shifts are larger. However, Milligan et al. focused on environmental changes no greater than the width of the fitness function (*Ω*), explicitly excluding scenarios where the climate shift is severe enough to reduce population sizes and risk extinction. Within this bounded regime, they focus on allele dynamics assuming constant population size and show that large-effect alleles are often selected against during the stabilizing selection phase after the early directional selection phase.

In contrast, fewer studies have explicitly examined the effect of genetic architecture in scenarios where populations would go extinct in the absence of rapid adaptation, that is, within the framework of evolutionary rescue. Gomulkiewicz et al. modeled adaptation to harsh environments and showed that a combination of a rare major-effect allele and polygenic background variation can enhance adaptation in a new environment (Gomulkiewicz et al. 2010). Their model predicted that adaptation driven solely by diffuse, small-effect loci may be too slow to enable persistence under strong selective shifts. Our results partially support this view: under severe environmental change, high-polygenic architectures alone often failed to produce the extreme phenotypes required for rescue (i.e. the phenotypic distribution did not overlap with the new fitness distribution), reinforcing the idea that polygenic architectures may have limited evolutionary potential. However, we think their model outcome is partly driven by their assumption of fixed *V_A_*(*w*) across genetic architectures at simulation onset, which can artificially dilute selection on polygenic traits. In contrast, our simulations allow trait distributions—and thus *V_A_*(*w*)—to emerge organically from the underlying genetic architecture, finding a higher average *V_A_*(*w*) in polygenic architectures across replicates but more extreme *V_A_*(*w*) values in monogenic architectures. A later key study showed that high polygenic architectures confer greater population viability during rapid environmental change, primarily due to their higher short-term evolutionary potential, which they attribute to a more stable maintenance of heritability (Kardos and Luikart 2021). However, our analyses showed both *V_A_* and *h*^2^ consistently declined through time across architectures, with the fastest losses occurring in low polygenic architectures (Supplementary Figs S1 and S2). Our findings align with this under mild-to-moderate environmental shifts, where polygenic traits consistently enhanced survival. Their definition of a new environmental optimum, 3.2 $\surd{} $*V_A_* from the initial phenotypic mean, falls within what we classify as small-to-intermediate change. In those scenarios we also observe better polygenic performance. Notably, Kardos and Luikart also observed high variability across low-polygenic architectures and emphasized that their outcomes were sensitive to initial allele frequencies, an observation that closely mirrors our own.

A key insight of our findings is that the environment interacts with the genetic architecture to create unexpected distributions of genetic variances in fitness. After Fisher’s Fundamental Theorem of Natural Selection, which equates *V_A_*(*w*) to the rate of adaptation, we found that average *V_A_*(*w*) positively correlated with the probability of evolutionary rescue across all our simulations (Fig. 4) (Fisher 1930). A fundamental observation from Fisher that led to integrating Mendelian genetics and biometrician views of inheritance was that as the number of Mendelian loci involved in a trait increases, the distribution of phenotypes becomes a Gaussian distribution with population variance *V_A_* (Fisher 1918). An obvious observation from first principles, and with critical importance in our study, is that genetic architectures of low numbers of loci (i.e. <30) generate phenotypic distributions that do not conform to Gaussian assumptions. In our study, we realized that nonnormality of phenotypic distribution has critical consequences for fitness variance. Even when simulations started with the same mean genetic value and variance (*V_A_*), the resulting distribution of *w* differed systematically across architectures (Figs 5 and 6). Low-polygenic architectures produced *V_A_*(*w*) with higher variance across replicated simulations (*Var*[*V_A_*(*w*)]_monogenic_ > *Var*[*V_A_*(*w*)]_polygenic_) and with significant skewness (*Skew*[*V_A_*(*w*)]_monogenic_ > 0, *Skew*[*V_A_*(*w*)]_polygenic_ ~ 0), leading to more volatile and unpredictable evolutionary rescue outcomes. Furthermore, the average *V_A_*(*w*) varied across environments such that under small environmental change, polygenic architectures led to on average higher (*E*[*V_A_*(*w*)|*∆*_small_]_polygenic_ > *E*[*V_A_*(*w*)|*∆*_small_]_monogenic_) but the opposite presented under more extreme environments (*E*[*V_A_*(*w*)|*∆*_big_]_monogenic_ > *E*[*V_A_*(*w*)|*∆*_big_] _polygenic_). A fixed *V_A_*(*w*) across architectures, as utilized in previous studies, prevented studying the interaction of environment and architecture shaping genetic variance. We hence suggest studying this phenomenon analytically in future work.

**Fig. 8:**
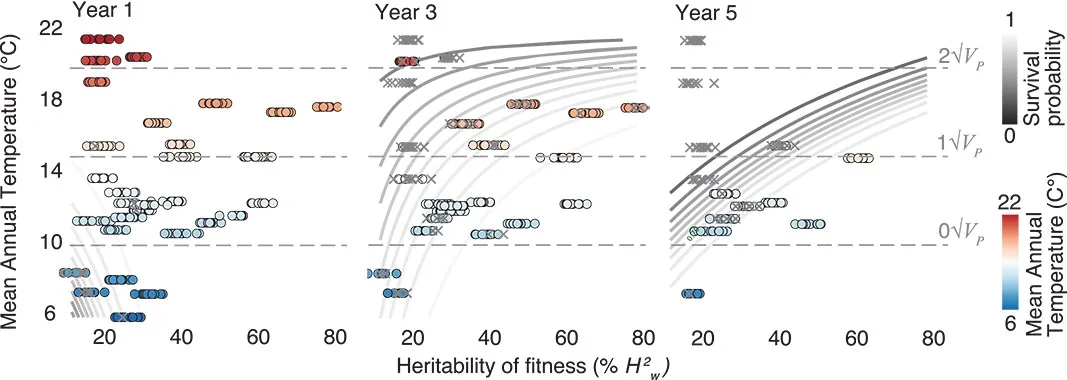
Interaction between environment and genetic variance in fitness in experimental evolution plots of *Arabidopsis thaliana*. Modified from Wu et al. (2026). *A. thaliana* populations were planted in field sites spanning a climate gradient. Circles represent surviving populations, and *X*s denote extinction. Survival probability was modeled using ecotype-composition predictability across environments and climate (mean annual temperature). Dashed horizontal lines denote the climate expressed as $\surd{} $*V_P_* of the ecotype’s climatic origin. *X*-axis values are jittered for clarity; each site has a single heritability estimate.

Contemporary conservation genetics has mainly focused on protecting genetic diversity within animal and plant species, to indirectly protect their long-term evolutionary potential (Allendorf et al. 2012). More recently, there have been calls to include genomic information toward anticipating (mal)adaptive conditions of populations based on their mismatch of locally adaptive genetic variation with future climate shifts (Capblancq et al. 2020). Our results suggest that a key dimension to understanding the (un)predictability of future evolutionary rescue is genetic architecture. Some recent studies have attempted to quantify, for instance, the heritability *H_w_*^2^ and *V_A_*(*w*) of fitness through pedigrees in wild populations to quantitatively assess adaptation potential. For instance, Bonnet et al. (2022) found *V_A_*(*w*) values significantly different from zero in 10 of 19 vertebrate natural populations, although the ratio to total fitness variance was small, *h*^2^*_w_* = 3% (Bonnet et al. 2022). Plant common garden experiments and tree provenance trials, in which genotypes from different source populations are compared together in the same environment, often show significantly larger heritabilities in fitness traits: *h*^2^ ~ 0% to 64% (Roybal and Butterfield 2018; Bemmels and Anderson 2019; Leventhal et al. 2025b). In *A. thaliana* common gardens, where fitness is often directly measured and used for GWAs, previous studies have found a highly polygenic architecture (Exposito-Alonso et al. 2019; Leventhal et al. 2025b; Wu et al. 2026). A recent experimental evolution study conducted in outdoor plots to investigate rapid adaptation and extinction in nature inspired our simulations (Wu et al. 2026). A key result of this experimental evolution study is the interaction of *V_A_*(*w*), the transplant environment, and long-term population survival (Fig. 8). Under extreme environmental shifts—such as the transplant distance of *A. thaliana* populations to a common garden calculated based on temperature of origin—a high *V_A_*(*w*) measured in the first year of the experiment was predictive of survival within 5 years. Such experimental approaches modifying the starting genetic composition of the population would be ideal to better understand the complexities of genetic architectures and adaptation in realistic environments.

Incorporating knowledge of genomic architecture’s influence on evolutionary rescue into conservation management could provide valuable insights. For example, the efficiency of assisted gene flow may be affected by the genetic architecture of adaptation in a species. Simulations have shown that adaptation can occur rapidly after the translocation of individuals carrying one or a few large-effect alleles (Grummer et al. 2022). In contrast, when adaptation depends on many small-effect alleles, the benefits may take much longer to manifest, as each allele contributes only a small portion of the adaptive response and thus takes longer to introgress into the local population (Grummer et al. 2022). Furthermore, as genomic technologies become more accessible in nonmodel species, it has become increasingly clear that local adaptation often involves clusters of tightly linked loci—blurring the line between monogenic and polygenic architectures (Schwander et al. 2014; Gutiérrez-Valencia et al. 2021). These so-called “supergenes” underlie color polymorphisms in butterflies (M. J. Thompson and Jiggins 2014) and ecotypic differentiation in sunflowers (Todesco et al. 2019). Although these traits are genetically complex, due to tight linkage, their evolutionary dynamics better approximate monogenic adaptation (Oomen et al. 2020; Yeaman 2022).

**Fig. 9:**
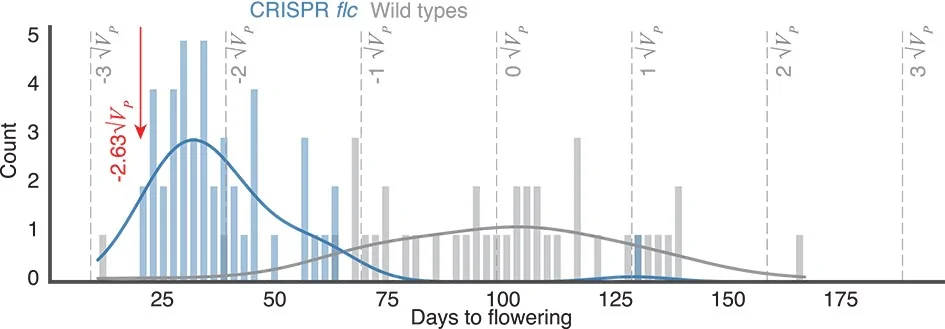
Distribution of mean flowering time per ecotype for wild-type (wt) and CRISPR *flc* knockout (KO) individuals across 94 *Arabidopsis* ecotypes. Modified from Ruffley et al. (2024). For each genotype, flowering time was averaged across three replicates. Vertical dashed lines show $\surd{} $*V_P_* of the wild-type distribution. The arrow marks the minimum flowering time observed among the flc KO lines, which lies 2.63 $\surd{} $*V_P_* units below the wt mean. All plants were grown under long-day (LD) conditions at 22°C.

Theoretically, if future work ever achieves sufficient understanding of the genetic basis and architecture of adaptation, it may even be possible to directly engineer adaptive traits through gene editing (Wang and Doudna 2023). This may be a last resort when within-species genetic diversity has been severely depleted—a scenario that is already a concern for many species (Exposito-Alonso et al. 2022). However, conservationists have rightly cautioned that uncertainty in identifying the correct traits or targets for intervention may result in ineffective or even harmful outcomes (Kardos and Shafer 2018). While current CRISPR/Cas9 technologies have advanced to the point that nearly *any* gene can be edited in plants and animals (Wang and Doudna 2023; Gilbertson et al. 2025), the challenge lies in identifying which genes to target. While we are far, if ever, from nature interventions, recent gene editing studies of few well-characterized traits—such as coat color in mice, recently modified to mimic that of woolly mammoths (Chen et al. 2025), or spring flowering time pathways in Brassicaceae, used to accelerate phenology in wild accessions (Lutz et al. 2025; Ruffley et al. 2024)—can serve as a thought experiment. Using again the model system *A. thaliana*, we can directly visualize the effect of a single mutation in a key adaptive trait, spring flowering time, by targeting the *FLOWERING LOCUS C* (*FLC*) gene. This mutation led to significantly earlier (≈60 days) flowering across 47 knock-out or knock-down lines engineered in 47 wild-type accession lines originally collected from different natural populations in the Northern range (Fig. 9) (Lutz et al. 2025; Ruffley et al. 2024). We estimated that such phenotypic shift corresponds to over >2$\surd{} $*V_P_*. Under these circumstances, if adaptation to global change was solely determined by accelerating flowering time, a large-effect loss-of-function *flc* mutation may catalyze evolutionary rescue. Yet this strategy becomes far more precarious in natural population conservation, where fitness is rarely determined by a single trait but rather emerges from the combined effect of many underlying traits, or where large-effect loci may be purged due to environmentally varying selection and stabilizing selection. As a result, fitness-related traits are typically highly polygenic, and their expression is further modulated by gene-by-environment interactions. From these principles, one may derive that gene editing could be used to generate genetic diversity in polygenic architectures; yet, while theoretically interesting, this is unlikely to be practical or well understood for the vast majority of species.

As anthropogenic environmental change accelerates, accurately predicting the probability, pace, and direction of evolutionary rescue is critical for biodiversity conservation and ecosystem management (Schoener 2011; Bay et al. 2017; Coulson et al. 2017; Forester et al. 2022). Advances in genomic technologies now make it increasingly possible to infer aspects of the genetic architecture underlying adaptive traits in natural populations and to estimate their adaptive potential, providing critical information for improving predictive models of population persistence and guiding conservation strategies (McLachlan et al. 2007; Allendorf et al. 2012; Funk et al. 2012; Thomas et al. 2013; Hoffmann et al. 2015; Shafer et al. 2015). Our results highlight the value of incorporating genetic architecture parameters into predictive frameworks, as they significantly influence both the likelihood and predictability of adaptive responses. In particular, populations where adaptation will depend on highly polygenic traits may offer predictability and greater resilience to moderate environmental shifts, but those depending on low-polygenic adaptive traits may occasionally achieve rescue under extreme change, albeit with higher stochastic risk.

## Supplementary Material

review2_Bellagio_2026_SupMat_Figures_esag026

## Data Availability

All code required to reproduce the analyses presented in this study is openly available at: https://github.com/Tatianabellagio/slim_grenenet/tree/es_dep_af.
