# Supplementary material for: Polygenic and monogenic adaptation drive evolutionary rescue at different magnitudes of environmental change
Source: J Hered. 2026 Apr 7;117(5):1073–86. doi: 10.1093/jhered/esag026 (PMC13539452; doi:10.1093/jhered/esag026)
Supplement: Supplementary file 1 [file review2_bellagio_2026_supmat_figures_esag026.pdf]

Supplemental Materials for Bellagio & Exposito-Alonso (2025)

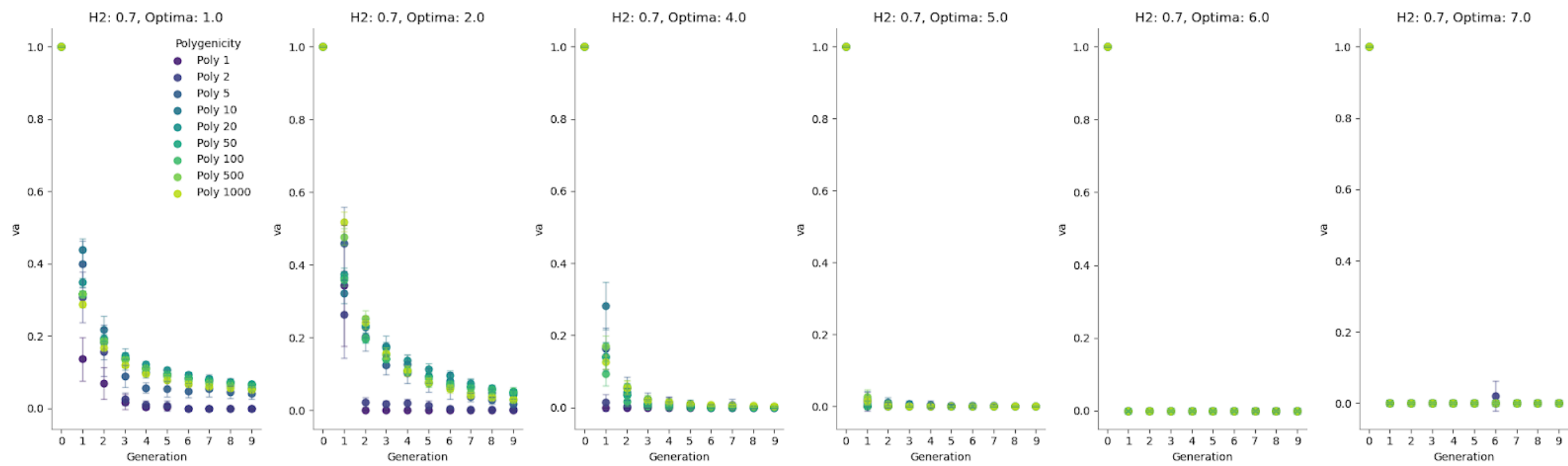

Supplemental Figure 1

Genetic variance of phenotype (Va) through time across genetic architectures and magnitudes of environmental shift, where simulations were started with h^2=0.7.

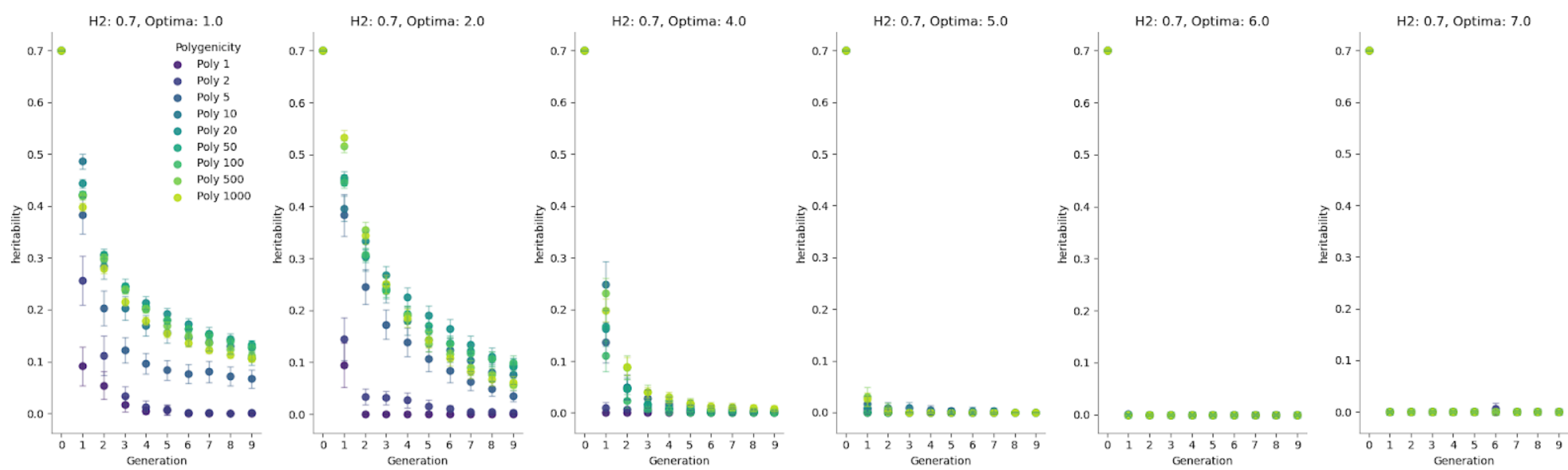

Supplemental Figure 2

Heritability decay through time across genetic architectures and magnitudes of environmental shift, where simulations were started with h^2=0.7.

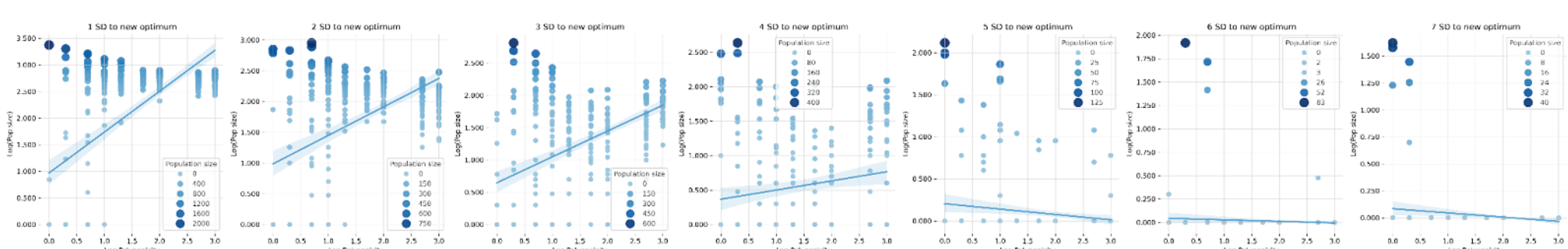

Supplemental Figure 3

Relationship between polygenicity and final population size (10th generation) under different magnitudes of environmental change. Simulations were initialized with h2=0.7.

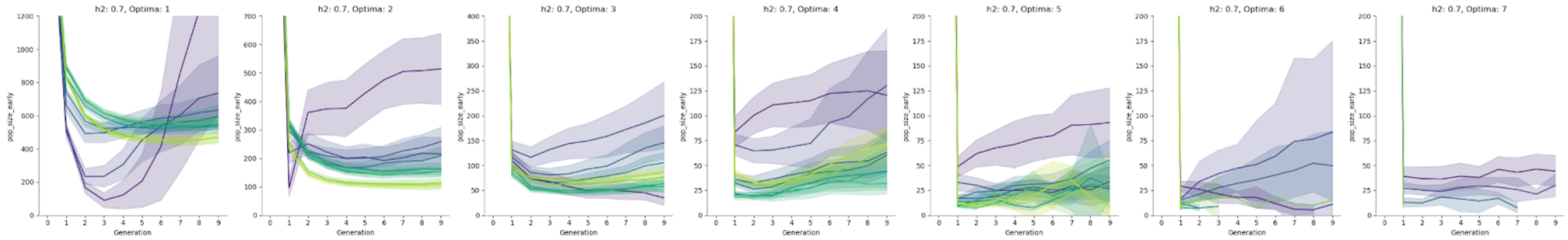

Supplemental Figure 4

Population trajectories across time under different polygenic architectures and environmental shift magnitudes. Simulations were initialized with h2=0.7.

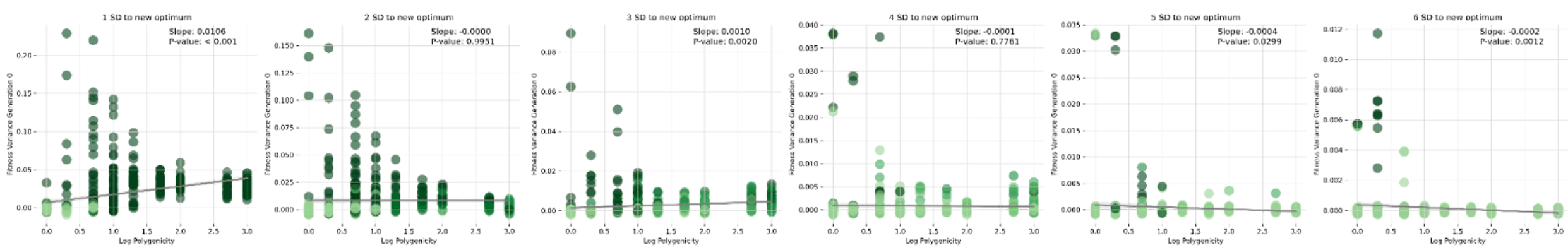

Supplemental Figure 5

The effect of polygenicity on additive genetic variance in fitness (VA(w)) under different magnitudes of environmental change. Simulations were initialized with h2=0.7.

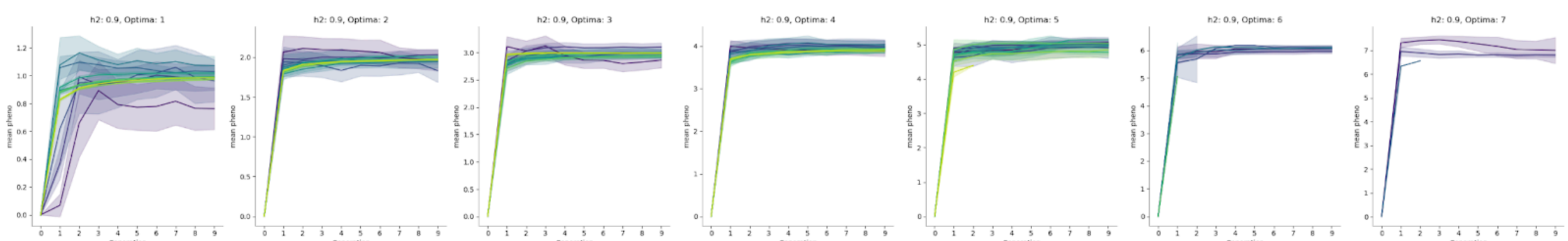

Supplemental Figure 6

Populations' mean phenotypic value change across generations under multiple levels of environmental shift, showing that surviving populations track the new phenotypic optimum. Simulations were initialized with h2=0.7.
